# Acetaldehyde-Mediated Neurotoxicity: Relevance to Fetal Alcohol Spectrum Disorders

**DOI:** 10.1155/2011/213286

**Published:** 2011-05-23

**Authors:** Ming Tong, Lisa Longato, Quynh-Giao/Ly Nguyen, William C. Chen, Amy Spaisman, Suzanne M. de la Monte

**Affiliations:** ^1^Department of Medicine, Rhode Island Hospital and the Alpert Medical School of Brown University, Providence, RI 02903, USA; ^2^Pathobiology Graduate Program, Brown University, Providence, RI 02912, USA; ^3^Departments of Pathology, Neurology, and Neurosurgery, Rhode Island Hospital and the Alpert Medical School of Brown University, Providence, RI 02903, USA

## Abstract

Ethanol-induced neuro-developmental abnormalities are associated with impaired insulin and IGF signaling, and increased oxidative stress in CNS neurons. We examined the roles of ethanol and its principal toxic metabolite, acetaldehyde, as mediators of impaired insulin/IGF signaling and oxidative injury in immature cerebellar neurons. Cultures were exposed to 3.5 mM acetaldehyde or 50 mM ethanol ± 4-methylpyrazole (4-MP), an inhibitor of ethanol metabolism, and viability, mitochondrial function, oxidative stress, DNA damage, and insulin responsiveness were measured 48 hours later. Acetaldehyde or ethanol increased neuronal death and levels of 8-OHdG and 4-HNE, and reduced mitochondrial function. Ethanol inhibited insulin responsiveness, whereas acetaldehyde did not. 4-MP abated ethanol-induced oxidative stress and mitochondrial dysfunction, but failed to restore insulin responsiveness. Furthermore, alcohol and aldehyde metabolizing enzyme genes were inhibited by prenatal ethanol exposure; this effect was mediated by acetaldehyde and not ethanol + 4MP. These findings suggest that brain insulin resistance in prenatal alcohol exposure is caused by direct effects of ethanol, whereas oxidative stress induced neuronal injury is likely mediated by ethanol and its toxic metabolites. Moreover, the adverse effects of prenatal ethanol exposure on brain development may be exacerbated by down-regulation of genes needed for metabolism and detoxification of alcohol in the brain.

## 1. Introduction

Prenatal alcohol exposure causes fetal alcohol spectrum disorder (FASD), which is associated with multiple and varied developmental abnormalities in the brain and results in sustained deficits in cognitive and motor functions [1–6]. Ethanol exerts its neurotoxic and teratogenic effects [7, 8] by promoting oxidative stress and impairing insulin and insulin-like growth factor (IGF) signaling in the developing brain [9, 10]. Whether these effects are mediated by direct toxic effects of ethanol or its principal metabolite, acetaldehyde, has not yet been determined.

Ethanol has broad inhibitory effects on insulin and IGF signaling in the developing brain and immature neurons. For example, ethanol impairs ligand-receptor binding, tyrosine phosphorylation and activation of receptor tyrosine kinases, transmission of signals through insulin receptor substrate (IRS) proteins, and downstream activation of phosphatidylinositol-3 kinase (PI3 kinase)-Akt, p21^ras^, and mitogen-activated protein kinase kinase (MAPKK) [11]. Consequences include reduced neuronal proliferation, survival, migration, mitochondrial function, ATP production, membrane integrity, plasticity, and neurotransmitter function [10, 12–23]. Importantly, ethanol impairs insulin/IGF signaling and impairs ligand-receptor binding [17], and it activates phosphatases that negatively regulate receptor tyrosine kinases (PTP-1b) [24–26]. Therefore, ethanol exposure causes major defects in insulin/IGF signaling, beginning at the most proximal points within the cascades, and effectively results in a state of chronic insulin/IGF resistance in developing CNS neurons. 

Ethanol also has cytotoxic effects that are manifested by increased oxidative stress and DNA damage. Ethanol toxicity perturbs the structural and functional integrity of mitochondria, particularly in liver and brain. Ethanol causes oxidative modification of mitochondrial DNA (MtDNA), manifested by increased 8-hydroxydeoxyguanosine (8-OHdG) incorporation, reduced MtDNA content, and increased MtDNA single-strand breaks [12, 23, 27–30]. Ethanol-induced MtDNA damage and impaired Mt function increase cellular sensitivity to toxins and promote Mt permeability transition resulting in necrosis or apoptosis [12, 23, 29, 30]. These adverse effects of ethanol are likely mediated by increased oxygen free radical production, lipid peroxidation, and inhibition of Mt glutathione. Ethanol metabolism by the microsomal monoxygenase system, involving the alcohol-inducible cytochrome P450 2E1 (CYP2E1), could contribute to oxidative cellular injury through hydroxylethyl radical formation [31, 32]. 

Alcohol is metabolized and detoxified by a series of oxidation reactions, beginning with reversible oxidation of ethanol to acetaldehyde by alcohol dehydrogenase (ADH), CYP2E1, and catalase [33–35]. However, ADH, which has a high affinity for alcohol, is the main oxidizing enzyme [36]. The cytosolic localization of ADH leads to acetaldehyde formation and accumulation in the cytoplasm. CYP2E1 is induced by chronic alcohol consumption and results in acetaldehyde formation in peroxisomes. Catalase, which is abundantly expressed in brain, oxidizes alcohol to acetaldehyde in microsomes [37, 38]. Acetaldehyde, which is highly toxic, is irreversibly oxidized to acetate by mitochondrial aldehyde dehydrogenase (ALDH) as well as CYP2E1, a microsomal acetaldehyde-oxidizing system that utilizes an NADPH-dependent pathway [39]. Activated acetate forms acetyl CoA, which breaks down to form CO_2_ and H_2_O [40]. 

Acetaldehyde accumulates and exerts its toxic effects when the enzymatic pathways responsible for oxidizing alcohol become overwhelmed. The electrophilic nature of acetaldehyde [36, 41] renders it highly reactive, enabling it to bind and form adducts, that is, covalent chemical additions, with proteins, lipids, and DNA [35, 42–45]. Adducts are pathogenic, because they impair functions of proteins and lipids, promote DNA damage and mutation [35], and increase the generation of reactive oxygen species (ROS) [14], resulting in broad-ranging impairments in protein function, gene expression, and DNA integrity, including increased mutagenesis [35, 45–47]. Increased levels of ROS can impair neuronal viability by inhibiting electron transport chain function and ATP formation [14, 48, 49].

## 2. Results

### 2.1. Acetaldehyde Impairs Neuronal Viability and Mitochondrial Function and Increases Oxidative Stress

**Figure 1:**
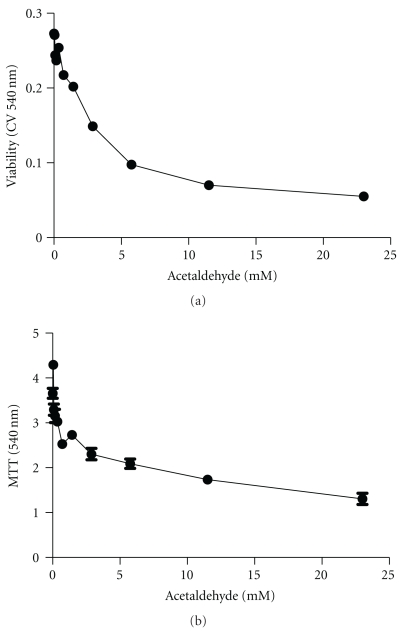
Acetaldehyde treatment impairs neuronal viability and mitochondrial function. Rat cerebellar neuron cultures were treated with 0.045–23.0 mM acetaldehyde for 48 hours. (a) Viability was measured using the Crystal violet assay, and (b) mitochondrial function was measured with the MTT assay. Graphs depict the mean ± S.E.M. of results from 16 replicate 96-well cultures.

 Initial studies characterized acetaldehyde dose effects on neuronal viability and mitochondrial function. Crystal violet staining was used to measure viability, and the 3-(4,5-Dimethylthiazol-2-Yl)-2,5-Diphenyltetrazolium Bromide (MTT) assay was used to measure mitochondrial function. Acetaldehyde exposure for 24 hours had modest effects on neuronal viability and mitochondrial function (data not shown). However, after 48 hours exposure, acetaldehyde resulted in dose-dependent declines in both cell viability and mitochondrial function (Figure 1). Subsequent experiments were conducted using 3.5 mM acetaldehyde treatment for 48 hours.

**Figure 2:**
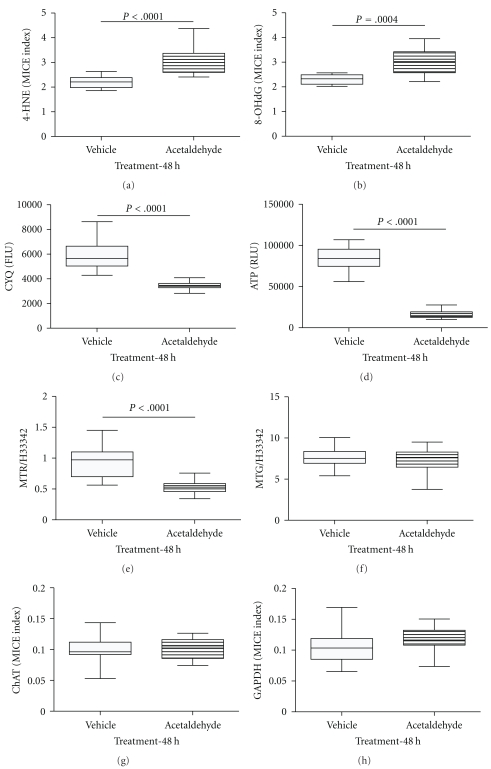
Acetaldehyde causes neuronal oxidative stress, DNA damage, and mitochondrial dysfunction. Rat cerebellar neuron cultures were treated with 3.5 mM acetaldehyde for 48 hours, and used to measure (a) 4-HNE, (b) 8-OHdG, (c) Cyquant fluorescence-viability (CYQ), (d) ATP content, (e) MitoTracker Red (MTR), (f) MitoTracker Green (MTG), (g) ChAT and (h) GAPDH. 4-HNE, 8-OHdG, ChAT, and GAPDH immunoreactivities were measured by cellular ELISA. Viability and mitochondrial assays were measured in labeled cells. All results were normalized to H33342 fluorescence, which is linearly correlated with cell number. Box plots depict the mean (horizontal bar), 95% confidence interval limits (upper and lower edges of boxes), and range (whiskers). Inter-group comparisons were made using Student T-tests. Significant differences are indicated within the panels.

Corresponding with the crystal violet assay results, the CyQuant assay demonstrated significantly reduced cell viability in acetaldehyde-treated cultures (Figure 2(c)). MitoTracker dye studies and measurements of ATP content were used to better characterize effects of acetaldehyde treatment on mitochondrial function. Acetaldehyde treatment significantly reduced MitoTracker Red (Figure 2(e)), but it did not significantly alter MitoTracker Green fluorescence (Figure 2(f)). In addition, ATP content was profoundly reduced by the acetaldehyde treatment (Figure 2(d)). 

Cellular enzyme-linked immunosorbent assays (ELISAs) were used to measure immunoreactivity corresponding to 4-hydroxy-2-nonenal (4-HNE), 8-hydroxydeoxyguanosine (8-OHdG), choline acetyltransferase (ChAT), and glyceraldehyde-3-phosphate dehydrogenase (GAPDH). The acetaldehyde-induced reductions in cell viability and mitochondrial function were associated with significantly increased levels of 4-HNE (Figure 2(a)) and 8-OHdG (Figure 2(b)) immunoreactivity, reflecting increased lipid peroxidation and DNA damage, respectively. In contrast, acetaldehyde treatment did not significantly alter ChAT or GAPDH immunoreactivity Figures 2(g) and 2(h), both of which are responsive to insulin and IGF-1 stimulation [50]. 

### 2.2. Acetaldehyde Does Not Impair Neuronal Insulin Signaling

**Figure 3:**
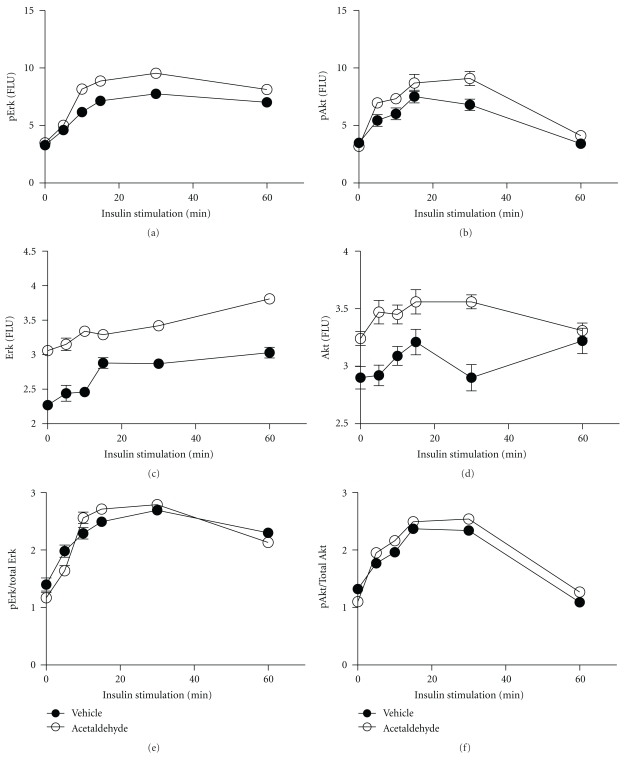
Acetaldehyde does not impair insulin stimulated signaling through ERK and Akt. Rat cerebellar neuron cultures seeded into 96-well plates, were treated with 3.5 mM acetaldehyde for 48 hours, and then stimulated with 10 nM insulin for up to 60 minutes. Cells were fixed and used to measure (a) pERK, (b) pAkt, (c) total ERK, and (d) total Akt by cellular ELISA, and results were normalized to H33342 fluorescence. The ratios of (e) pERK/total ERK and (f) pAkt/total Akt were calculated to show relative time-dependent shifts in insulin-stimulated signaling. Graphs depict the mean ± S.E.M. of results generated from 8 replicate assays. These results are strikingly different from those obtained using ethanol-treated cultures (see Figure 4). Inter-group differences with respect to pERK, pAkt, ERK, and Akt are statistically significant by area-under-curve analysis. However, relative levels of ERK and Akt phosphorylation are similar for vehicle and acetaldehyde-treated cultures.

**Figure 4:**
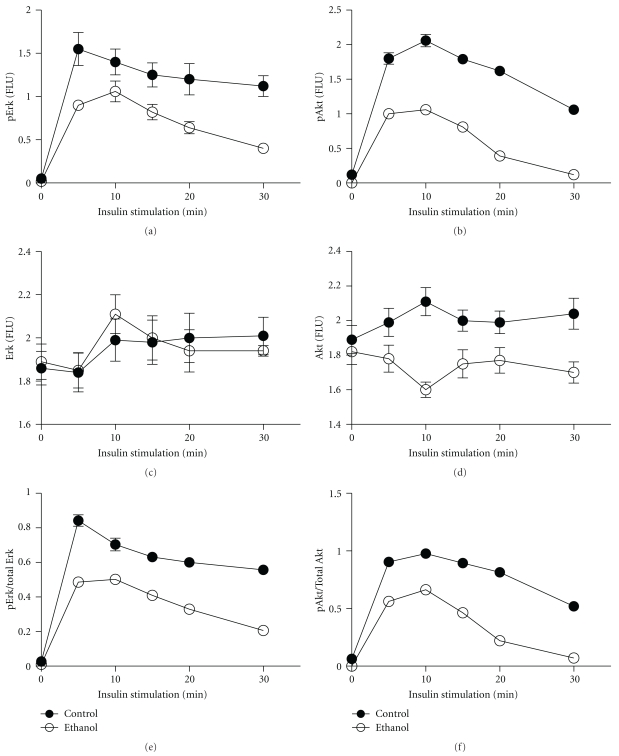
Ethanol impairs insulin-stimulated phosphorylation of ERK MAPK and Akt. Control and ethanol-exposed (50 mM x 48 h) rat cerebellar neuron cultures, seeded into 96-well plates, were stimulated with 10 nM insulin for up to 30 minutes. Cells were fixed and used to measure (a) pERK, (b) pAkt, (c) total ERK, and (d) total Akt by cellular ELISA, and results were normalized to H33342 fluorescence. The ratios of (e) pERK/total ERK and (f) pAkt/total Akt were calculated to show relative time-dependent shifts in insulin-stimulated signaling. Graphs depict the mean ± S.E.M. of results generated from 8 replicate assays. Inter-group differences with respect to pERK, pAkt, pERK/Total ERK, and pAkt/Total Akt are statistically significant by area-under-curve analysis.

 In previous studies, we demonstrated that acute or chronic exposure to ethanol leads to impaired insulin-stimulated responses in CNS neuronal cells, resulting in significant inhibition of insulin-stimulated phosphorylation of ERK MAPK and Akt [10]. To determine if these effects could be mediated by acetaldehyde, 96-well acetaldehyde (3.5 mM) or vehicle treated (48 h) microcultures were stimulated with 10 nM insulin for up to 60 minutes, after which they were fixed in situ and examined for immunoreactivity to pERK, total ERK, pAkt, or total Akt by cellular ELISA. Immunoreactivity was normalized to H33342 fluorescence, and relative increases in phosphorylation, that is, phospho/total protein, were calculated for each time point. Eight replicate assays were performed per time point, and the experiments were repeated twice (Figure 3). The results demonstrated higher levels of pERK, ERK, pAkt, and Akt in acetaldehyde-treated relative to control at nearly all time points (Figures 3(a)–3(d)). However, acetaldehyde and vehicle-treated cells exhibited similar trends with regard to the time course of insulin-stimulated pERK (Figure 3(a)) and pAkt (Figure 3(b)), and curves defining the calculated phospho/total protein ratios were virtually identical for both kinases (Figures 3(e) and 3(f)). This result contrasts with the effects of ethanol, which significantly reduced insulin-stimulated phospho- and phospho/total ERK and Akt levels at all time points (Figure 4). 

### 2.3. Chronic Prenatal Ethanol Exposure Alters Alcohol Metabolizing Enzyme Gene Expression in Brain

 The in vitro experiments demonstrate that acetaldehyde exposure has profound inhibitory effects on viability and mitochondrial function and that it increases oxidative stress and DNA damage in immature CNS neurons. To determine the potential role of acetaldehyde-mediated neurotoxicity as a mediator of neurodevelopmental defects in FASD, it was important to characterize the expression profiles of genes that regulate alcohol and acetaldehyde metabolism and determine the effects of ethanol exposure on the expression of these genes. We used qRT-PCR analysis to measure expression of multiple isoforms of alcohol and acetaldehyde dehydrogenase, in addition to CYP2E1 and catalase. Those initial studies demonstrated that ADH1, ADH7, ALDH1, ALDH2, ALDH3, CYP2E1, and catalase mRNA transcripts were detectable in normal rat pup cerebella. Subsequent studies were narrowed to examine effects of chronic gestational exposure to ethanol on the expression of these 7 genes. 

**Figure 5:**
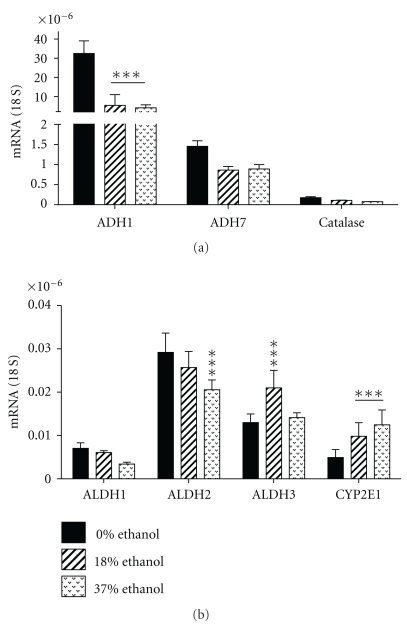
Effects of prenatal ethanol exposure on expression of alcohol metabolizing enzyme gene expression in cerebellar tissue. Cerebella from postnatal day 2-3 rat pups from dams that had been chronically fed with isocaloric liquid diets containing 0%, 18%, or 37% ethanol by caloric content were used to measure expression of different isoforms of (a) alcohol dehydrogenase (ADH) or (b) aldehyde dehydrogenase (ALDH), as well as catalase and CYP2E1 by qRT-PCR analysis. Results were normalized to 18S rRNA measured in the same samples. Inter-group comparisons were made using repeated measures ANOVA with the Dunnett post hoc test to detect significant differences from control. *P < .05; ***P < .01.

The in vivo experimental models were generated by feeding pregnant Long Evans rat dams with isocaloric liquid diets that contained 0%, 18%, or 37% ethanol by caloric content as previously described. Rat pups exposed to the 18% ethanol diet had moderate abnormalities in cerebellar architecture, while those exposed to the 37% ethanol diet had fetal alcohol syndrome with severe cerebellar hypoplasia and pronounced deficits in neuronal migration [17, 51]. The gene expression studies demonstrated that chronic gestational exposure to the 18% or 37% ethanol containing diets significantly reduced expression of ADH1, ALDH2, and catalase, and significantly increased expression of CYP2E1 (Figure 5). In addition, exposure to the 37% diet significantly reduced ALDH1 expression, while exposure to the 18% ethanol diet significantly increased ALDH3 expression. Inhibition of ADH and catalase would lead to increased local levels of ethanol, whereas inhibition of ALDH and CYP2E1 would result in increased local levels of acetaldehyde. 

### 2.4. Ethanol's Toxic Metabolites Cause Oxidative Stress and Mitochondrial Dysfunction, but Not Insulin Resistance

**Figure 6:**
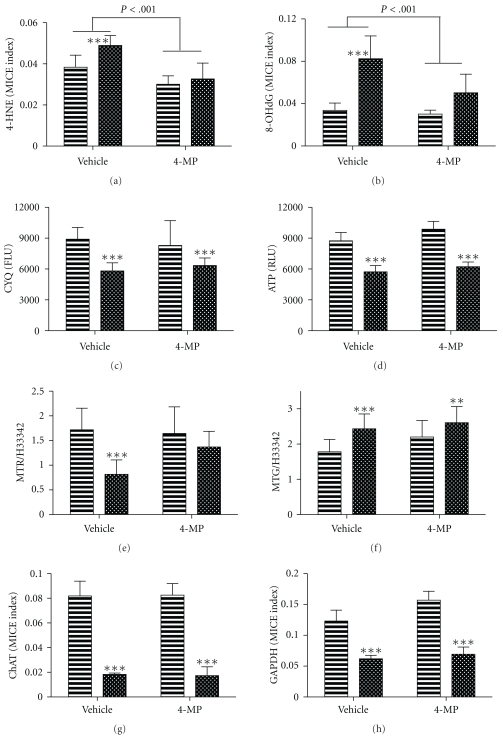
Effects of 4-methylpyrazole (4-MP) treatment on ethanol-induced neurotoxicity. Control or ethanol exposed (50 mM) rat cerebellar neuron cultures seeded into 96-well plates were treated with vehicle or 4-MP for 48 h. The cells were used to measure (a) 4-HNE, (b) 8-OHdG, (c) Cyquant fluorescence-viability (CYQ), (d) ATP content, (e) MitoTracker Red (MTR), (f) MitoTracker Green (MTG), (g) ChAT, and (h) GAPDH. 4-HNE, 8-OHdG, ChAT, and GAPDH immunoreactivities were measured by cellular ELISA. Viability and mitochondrial assays were measured in labeled cells. All results were normalized to H33342 fluorescence. Graphs depict the mean ± S.D. of results. Statistical comparisons were made using two-way ANOVA with the post hoc Bonferroni significance test. Significant differences are shown within each panel. **P < .01 and ***P < .001 between control and ethanol-exposed cells within the vehicle- or 4-MP treated groups.

We next performed experiments to determine the degree to which oxidative injury, DNA damage, mitochondrial dysfunction, and impaired insulin responsiveness were mediated by ethanol or its toxic metabolites. Neuronal cultures (96-well) were exposed to 50 mM ethanol for 96 h and treated with vehicle or 4-MP. The cultures were stimulated with 10 nM insulin for the last 24 h in culture. The ethanol-exposed, vehicle-treated cells had significantly higher levels of 4-HNE (Figure 6(a)), 8-OHdG (Figure 6(b)), and MitoTracker Green fluorescence (Figure 6(f)), and reduced levels of viability (Cyquant assay; Figure 6(c)), ATP production (Figure 6(d)), and mitochondrial function (MitoTracker Red fluorescence; Figure 6(e)) relative to corresponding controls. In addition, ChAT and GAPDH immunoreactivity were significantly reduced in the ethanol-treated cultures (Figures 6(g) and 6(h)). Simultaneous treatment with 4-MP significantly reduced 4-HNE immunoreactivity in both control and ethanol-exposed cultures. Otherwise, mitochondrial function, viability, and insulin-stimulated protein expression were not significantly changed by the 4-MP treatments. In contrast, 4-MP treatment of ethanol-exposed cultures significantly reduced 8-OHdG immunoreactivity and increased MitoTracker Red fluorescence such that the difference from control was no longer statistically significant. However, 4-MP treatment of ethanol-exposed cells did not significantly increase neuronal viability, ATP levels, or ChAT and GAPDH immunoreactivity. Therefore, the main positive effects of inhibiting ethanol metabolism with 4-MP were to reduce oxidative stress, DNA damage, and mitochondrial dysfunction.

## 3. Discussion

In FASD, the main focus has been on the role of ethanol-mediated neurotoxicity and the functional abnormalities leading to impairments in neurodevelopment. In particular, our studies have detailed the adverse effects of ethanol on insulin and IGF signaling mechanisms in the brain and CNS neuronal cells and demonstrated how impaired insulin/IGF responsiveness correlated with decreased neuronal survival, mitochondrial function, energy metabolism, migration, and neurotransmitter function [11]. However, those studies consistently correlated ethanol-associated neurodevelopmental structural and functional CNS abnormalities with impairments in trophic factor signaling, including insulin and IGF, and increased levels of oxidative stress. Since insulin signaling mediates metabolic functions, impaired insulin signaling or insulin resistance could reduce energy metabolism and promote oxidative stress. On the other hand, acetaldehyde, a major toxic metabolite of ethanol, is a potent mediator of oxidative stress due to ROS generation, increased lipid peroxidation and DNA damage, and formation of adducts that potentially could impair a broad array of cellular functions [33]. Therefore, it was of interest to determine the degree to which acetaldehyde accumulation is likely to contribute to CNS neuronal abnormalities in FASD.

Initial studies demonstrated that acetaldehyde is neurotoxic to immature cerebellar neurons and that it impairs both viability and mitochondrial function in a dose-dependent manner. The steep declines in viability and mitochondrial function observed following exposure to 0.045 to 0.72 mM acetaldehyde point to the marked sensitivity of CNS neurons to acetaldehyde, since previous studies demonstrated that exposures up to 20 mM or even 100 mM acetaldehyde were used to achieve cellular injury including DNA damage in vitro [52, 53]. The more detailed studies of acetaldehyde effects on neuronal viability and function were conducted with relatively low concentrations of acetaldehyde (3.5 mM) to avoid producing global severe injury that would cause extensive neuronal cell death.

Using in vitro functional assays combined with cellular ELISAs, we demonstrated that acetaldehyde treatment significantly increased 4-HNE and 8-OHdG immunoreactivity, reflecting lipid peroxidation and DNA damage with adduct formation in immature neuronal cells. Previous studies demonstrated that these effects are characteristic of acetaldehyde exposure in other cell types, including hepatocytes [33]. Moreover, comparing the effects of ethanol versus ethanol + 4-MP treatment, it appears that acetaldehyde rather than ethanol mediates these effects in ethanol-exposed neuronal cells. In essence, accumulation of acetaldehyde in the setting of CNS ethanol exposure and metabolism may be largely responsible for the attendant lipid peroxidation, permanent DNA damage, and generation of ROS. In contrast, neuronal viability (CyQuant assay) and ATP production were strikingly reduced by acetaldehyde and ethanol ± 4-MP treatment, suggesting that these functions are significantly compromised by ethanol and its main toxic metabolite, acetaldehyde. Importantly, inhibition of ethanol or acetaldehyde metabolism could result in higher local levels of either one or both, and thereby could lead to reduced neuronal viability and energy metabolism in immature CNS neurons.

MitoTracker fluorescent dye labeling studies were used to examine effects of acetaldehyde treatment on mitochondrial function (MitoTracker Red) and mitochondrial mass (MitoTracker Green). Previous studies revealed that ethanol impairs mitochondrial function in cerebellar neurons [10–13, 54]. The present work confirms that result and demonstrates prominent inhibitory effects of acetaldehyde on mitochondrial function but with no significant effect on mitochondrial mass. In addition, the studies demonstrated that ethanol exposure results in increased mitochondrial mass, which could serve to compensate for the impairments in mitochondrial function caused by mitochondrial DNA damage and inhibition of electron transport chain enzyme activity. The finding that 4-MP increased the MitoTracker Red fluorescence in ethanol-exposed cultures supports the notion that the mitochondrial dysfunction associated with ethanol exposure is largely mediated by acetaldehyde generation and accumulation in neuronal cells.

A major CNS abnormality associated with ethanol exposure, both during development and in the adult brain, is neuronal insulin resistance [10, 17, 24, 55–57], which is mediated by impaired ligand-receptor binding, activation of receptor tyrosine kinases, transmission of signals through IRS proteins, and activation of downstream pathways that promote neuronal survival, energy metabolism, plasticity, migration, and neurotransmitter function [50]. Previous studies suggested that oxidative stress can have either stimulatory [58] or inhibitory [59, 60] effects on insulin/IGF signaling through pathways that regulate cell migration, survival, and energy metabolism. Therefore, it was of interest to determine if acetaldehyde plays a role in dysregulating insulin signaling in the CNS. Herein, we measured insulin-stimulated ERK and Akt phosphorylation/activation in acetaldehyde-treated or ethanol-treated cerebellar neuron cultures. The results showed that acetaldehyde had no significant effect on the time course or levels of Erk or Akt phosphorylation relative to vehicle-treated cultures, whereas the ethanol treatments significantly impaired insulin-stimulated phosphorylation of Erk MAPK and Akt. Correspondingly, acetaldehyde treatment did not inhibit expression of ChAT and GAPDH proteins, and ethanol inhibition of ChAT and GAPDH was not prevented by 4-MP treatment. These results suggest that the adverse effects of ethanol leading to impairments in cholinergic function and neuronal migration are more likely due to inhibition of insulin/IGF signaling than the generation or accumulation of acetaldehyde in the brain. Ethanol impairs insulin signaling, in part by altering the plasma membrane lipid composition, leading to reduced ligand binding to receptors [17]. Although acetaldehyde exposure could also compromise growth factor signaling by forming adducts with cytoskeletal proteins and thereby impairing receptor-mediated endocytosis [60], the dominant effect of acetaldehyde is to form stable adducts with proteins, impairing their functions, and thereby promoting cell injury [35].

Finally, it was of interest to determine if chronic ethanol exposure itself renders the developing brain more susceptible to injury by alcohol or its metabolites. Gene expression studies demonstrated that chronic gestational exposure to ethanol inhibited ADH1, ALDH1, ALDH2, and catalase but increased CYP2E1 expression. Inhibition of ADH and catalase could result in higher local levels of ethanol, and thereby contribute to the inhibitory effects of ethanol on insulin/IGF signaling in the brain. In addition, inhibition of ALDH2 could promote acetaldehyde accumulation and lead to increased oxidative injury, lipid peroxidation, DNA damage, and ROS generation. The significantly increased levels of ALDH3 in the 18% ethanol group relative to control suggest a possible compensatory response to the inhibition of ALDH2. The greater impact of the higher levels of chronic ethanol exposure could be attributed in part to the simultaneous inhibition of ALDH1 and relatively normal (nonelevated) levels of ALDH3. The increased CYP2E1 expression observed in chronic ethanol-exposed brains is consistent with previous data showing that CYP2E1 is induced by chronic ethanol exposure [33]. However, consequences of increased CYP2E1 activity include excess ROS production, leading to increased oxidative stress and ROS-generated radicals including superoxide anion and hydroxyethyl radicals. In addition, CYP2E1-generated hydrogen peroxide in peroxisomes can react with metal ions to produce hydroxyl radicals. The net result is increased formation of adducts with lipids, proteins, and DNA, with attendant failure of a broad range of biological systems. 

## 4. Materials and Methods

### 4.1. Materials

The bicinchoninic acid (BCA) kit to measure protein concentration was purchased from Pierce Chemical Co. (Rockford, Ill). Histochoice fixative was purchased from Amresco, Inc. (Solon, Ohio). Amplex UltraRed soluble fluorophore, MitoTracker Red, MitoTracker Green, H33342, and CyQuant reagent were purchased from Invitrogen (Carlsbad, Calif). Maxisorp 96-well plates used for ELISAs were from Nunc (Thermo Fisher Scientific; Rochester, NY). Superblock-TBS, and horseradish peroxidase conjugated antibodies were from Pierce Chemical Co. (Rockford, Ill). Antibodies to active [pS473]-Akt and [pT202+pY204]-ERK1/ERK2 and total Akt and ERK MAPK were purchased from Cell Signaling Technology (Danvers, Mass). All other monoclonal antobodies and immunodetection reagents used for enzyme-linked immunosorbent assays (ELISAs) were purchased from Abcam (Cambridge, Mass), Upstate (Billerica, Mass), Vector Laboratories (Burlingame, Calif), Invitrogen (Carlsbad, Calif) or Chemicon (Temecula, Calif). Fine chemicals were purchased from CalBiochem (Carlsbad, Calif), or Sigma-Aldrich (St Louis, Mo). QIAzol Lysis Reagent for RNA extraction and QuantiTect SYBR Green PCR Mix were obtained from Qiagen, Inc. (Valencia, Calif). The AMV 1st Strand cDNA Synthesis Kit was purchased from Roche Applied Science (Indianapolis, Ind). Synthetic oligonucleotides used in quantitative polymerase chain reaction (qPCR) assays were purchased from Sigma-Aldrich Co. (St. Louis, Mo). ATPLite reagents were purchased from PerkinElmer (Boston, Mass). 

### 4.2. In Vitro Models

Primary neuronal cultures were generated with postnatal day 8 rat pup cerebella [24, 61] and maintained with Dulbecco's modified Eagle's medium supplemented with 5% fetal calf serum, 4 mM glutamine, 10 mM nonessential amino acid mixture (Gibco-BRL, Grand Island, NY), 25 mM KCl, and 9 g/L glucose. For ethanol treatment, 6-well or 96-well cultures were placed in sealed humidified chambers in which 50 mM ethanol was supplied to both the culture medium and reservoir tray [62]. Control cultures were identically treated but with only water added to the reservoir tray. The chambers were flushed with gas containing 75% nitrogen, 20% oxygen, and 5% carbon dioxide, and the medium was changed daily with fresh additions of ethanol to both the medium and reservoir tray. Cultures were incubated in the chambers for up to 96 hours at 37°C. To measure responsiveness to growth factor stimulation, the cells were serum-starved for 12 hours (starting after 48 hrs in the chambers) and then stimulated with 10 nM insulin or vehicle in the chambers for 48 hrs. To determine the effects of inhibiting ethanol metabolism on viability, mitochondrial function, and gene expression, the cultures were simultaneously treated with 4-methylpyrazole (4-MP). To examine effects of acetaldehyde exposure, 6-well or 96-well cultures (not maintained in chambers or ethanol exposed) were treated with up to 3.5 mM acetaldehyde for 48 h.

### 4.3. Cell Viability and Mitochondrial Studies

Cells seeded in 96 well plates were used to measure viability, mitochondrial mass, and mitochondrial function. Cell viability was measured using the crystal violet assay [63], and mitochondrial function was measured using the 3-(4,5-Dimethylthiazol-2-Yl)-2,5-Diphenyltetrazolium Bromide (MTT) assay [13]. MitoTracker Green FM (Ex490/Em516), which labels mitochondria irrespective of oxidative activity, was used to measure mitochondrial mass. MitoTracker Red (CM-H_2_XRos; Ex578/Em599), which accumulates in metabolically active mitochondria and is rendered fluorescent via oxidation within the mitochondria, was used to measure mitochondrial function. Cultures were labeled with cell permeable MitoTracker dyes for 15 minutes at 37°C according to the manufacturer's protocol, and fluorescence light units (FLUs) were measured with a Spectramax M5 microplate reader (Molecular Dynamics, Inc. Sunnyvale, Calif) [13, 54, 64]. To assess cell density, the cells were subsequently stained with Hoechst H33342 and fluorescence was measured (Ex 360 nm/Em 460 nm) in a Spectramax M5 [54, 64]. Results are expressed as MitoTracker/H33342 ratios calculated for 16 replicate cultures. ATP was measured using the ATPlite assay according to the manufacturer's protocol. Luminescence was measured in a TopCount machine (Packard Instrument Co).

### 4.4. Cellular Enzyme-Linked Immunosorbent Assay (ELISA)

A cellular ELISA was used to measure immunoreactivity directly in cultured cells (96-well plates) [65]. After overnight fixation in Histofix, cells were permeabilized with 0.05% Tween 20 in Tris-buffered saline, pH 7.5, then treated with 0.3% H_2_O_2_ in 60% methanol to quench endogenous peroxidase, and blocked with SuperBlock-TBS to adsorb non-specific binding sites. Cells were incubated over night at 4°C with 0.5–1 *μ*g/mL primary antibody. Immunoreactivity was detected with horseradish peroxidase conjugated Amplex Red soluble fluorophor, and fluorescence intensity (Ex 530/Em 590) was measured in a Spectramax M5 microplate reader (Molecular Dynamics, Inc., Sunnyvale, Calif). To assess cell density, cells were then labeled with Hoechst H33342 (Molecular Probes, Eugene, Ore) and fluorescence intensity (Ex360 nm/Em460 nm) was measured in the M5 Spectromax. Immunoreactivity was normalized to H33342 fluorescence. At least 8 replicate cultures were analyzed in each experiment. All experiments were repeated 3 times.

### 4.5. In Vivo Ethanol Exposure Model

Pregnant Long-Evans rats were pair-fed isocaloric liquid diets (BioServ, Frenchtown, NJ) containing 0%, 18%, or 37% ethanol by caloric content, or 0%, 4.5%, or 9.25% v/v ethanol [12, 17]. The liquid diets were begun on gestation day 6 and continued until parturition. This approach was used, because earlier periods of in utero ethanol exposure lead to excessive fetal loss due to impaired placentation [66]. Rats were monitored daily to ensure equivalent caloric consumption and maintenance of body weight. Cerebella harvested on postnatal days 2-3 were snap frozen in a dry ice-methanol bath and then stored at −80°C for later extraction of RNA to measure expression of alcohol metabolizing enzymes by qRT-PCR analysis. Cerebellar tissue was studied, because it represents a major target of ethanol neurotoxicity [2, 3, 24, 67, 68]. The Lifespan-Rhode Island Hospital IACUC committee approved these procedures and the use of rats in experiments.

### 4.6. RNA Studies

Total RNA was isolated from cultured cells or cerebellar tissue using the EZ1 RNA Universal Tissue Kit and the BIO Robot EZ1 (Qiagen Inc., Valencia, Calif). RNA was reverse transcribed with random oligonucleotide primers and the AMV First Strand cDNA synthesis kit, and the resulting cDNAs were used to measure gene expression by qPCR with gene-specific primer pairs (Supplementary Table 1 which could be found at doi: 10.1155/2011/213286). Primers were designed using MacVector 10 software (MacVector, Inc., Cary, NC), and target specificity was verified using NCBI-BLAST (Basic Local Alignment Search Tool). The Master ep realplex instrument and software (Eppendorf AG, Hamburg, Germany) were used to detect amplified signals from triplicate reactions. Relative mRNA abundance was calculated from the ng ratios of mRNA to 18S rRNA measured in the same samples, and those data were used for intergroup comparisons. Control studies included analysis of (1) template-free reactions, (2) RNA that had not been reverse transcribed, (3) RNA samples pretreated with DNAse I, (4) samples treated with RNAse A prior to the reverse transcriptase reaction, and (5) genomic DNA.

### 4.7. Statistical Analyses

Data depicted in bar graphs represent the means ± SEMs for each group. Data depicted in box plots reflect the median horizontal bar), 95% confidence interval limits (upper and lower boundaries of boxes), and range (whiskers). Intergroup comparisons were made using one- or two-way analysis of variance (ANOVA) with the Bonferroni post hoc test for significance. Ethanol dose-related trends were calculated by nonparametric linear regression analysis. Statistical analyses were performed using GraphPad Prism 5 software (GraphPad Software, Inc., San Diego, Calif). Significant *P* values (*P* < .05 or better) are indicated within the graph panels. 

## Supplementary Material

Table 1 legend: Primer pairs used for qRT-PCR analysis. ALDH = aldehyde dehydrogenase; ADH = alcohol dehydrogenase; F=forward primer; R=reverse primer; Position = binding site; Amplicon-PCR product size in bp
