# Supplementary material for: Acetaldehyde-Mediated Neurotoxicity: Relevance to Fetal Alcohol Spectrum Disorders
Source: Oxid Med Cell Longev. 2011 May 23;2011:213286. doi: 10.1155/2011/213286 (PMC3166768; doi:10.1155/2011/213286)
Supplement: Supplementary file 1 — Table 1 legend: Primer pairs used for qRT-PCR analysis. ALDH = aldehyde dehydrogenase; ADH = alcohol dehydrogenase; F=forward primer; R=reverse primer; Position = binding site; Amplicon-PCR product size in bp [file 213286.f1.docx]

**Supplementary Table 1: Primer pairs for quantitative RT-PCR analysis***

| RNA Target | Direction | Primer Sequence 5’ 🡪 3’ | Binding position | Amplicon Size (bp) |
| --- | --- | --- | --- | --- |
| 18S | Forward | GGA CAC GGA CAG GAT TGA CA | 1278-1327 | 50 |
| 18S | Reverse | ACC CAC GGA ATC GAG AAA GA | 1327-1278 |  |
| ALDH1 | Forward | ATC TGC CAT GT GGA AGA AGG | 173-193 | 216 |
| ALDH1 | Reverse | CAA GTA CGC ATT GGC AAA GA | 388-368 |  |
| ALDH2 | Forward | GAC CTG GAC AAG GCC AAT TA | 1391-1411 | 193 |
| ALDH2 | Reverse | TCT TCT GTG GCA CTT TGA CG | 1583-1563 |  |
| ALDH3 | Forward | CTG ATT GCT GAG GTT CCT GTT AGG | 1914-1937 | 119 |
| ALDH3 | Reverse | GGA TGT TTA GAC TGA GAG CCG ACT C | 2032-2008 |  |
| ADH1 | Forward | TGC TCC GTG CTG GAA AGA GTA TCC | 1138-1161 | 117 |
| ADH1 | Reverse | TAA GGT TGT GAT GTG GCT GGC G | 1254-1233 |  |
| ADH6 | Forward | TGT TGC CGA GTG AAT GAA AGG | 1155-1176 | 123 |
| ADH6 | Reverse | TGT TGC CGA GTG AAT GAA AGG | 1277-1257 |  |
| ADH4 | Forward | AGA GAG TGT GTC TGA TTG GAT GTG G | 585-609 | 163 |
| ADH4 | Reverse | AGA GAG TGT GTC TGA TTG GAT GTG G | 747-725 |  |
| ADH7 | Forward | CAA TGC TGC TTT TCA CTG GA | 971-991 | 234 |
| ADH7 | Reverse | AGA ACA CCC AGC TCT CTG GA | 1204-1184 |  |

*Primer pairs used in qRT-PCR analyses to measure mRNA levels of different isoforms of aldehyde and alcohol dehydrogenases. 18S rRNA was amplified in parallel reactions to normalize gene expression levels to total RNA input. ALDH = aldehyde dehydrogenase; ADH = alcohol dehydrogenase; Amplicon- PCR product size in base pairs (bp)
